# Clinical Lectures on Certain Acute Diseases

**Published:** 1860-04

**Authors:** 


Clinical Lectures on Certain Acute Diseases. By Robert Bentley Todd, M. D., F. R. S., Author of Lectures on Diseases of the Urinary Organs, etc., formerly Physician, now Consulting Physician to King's College Hospital, London. Philadelphia: Blanchard & Lea. 1860.

This is an octavo volume of 308 pages, occupied with fourteen Lectures; three of which are devoted to a consideration of Rheumatic Fever; two to Continued Fever; two to Erysipelas; one to Rheumatic Pericarditis and Endocarditis; one to Pyemia; four to Pneumonia; and one to the Therapeutical Action of

Alcohol. The general pathological and therapeutical doctrines advocated in the work, are distinctly shadowed forth in the following extract from the preface:

"The design of the lectures published in the present volume, is to describe and illustrate by examples the clinical history and treatment of the more important acute diseases.

There will (the author believes) be found in the following pages evidence enough to show that the ordinary so-called antiphlogistic treatment is unnecessary (to say the least) for the cure of acute internal inflammations; and that the supposed necessity for such treatment rested upon an untenable hypothesis respecting the nature of inflammation and of fevers, and cannot be regarded as a legitimate induction from accurately observed clinical facts.

The conclusions, which the clinical observations detailed in the lectures tend more or less to establish, may be summed up in the following propositions:—

1. That the notion so long prevalent in the schools, that acute disease can be prevented or cured by means which depress and reduce vital and nervous power, is altogether fallacious.

2. That acute disease is not curable by the direct influence of any form of drug or any known remedial agent, excepting when it is capable of acting as an antidote, or of neutralizing a poison, on the presence of which in the system the disease may depend (*materies morbi*).

3. That disease is cured by natural processes, to promote which, in their full vigor, vital power must be upheld. Remedies, whether in the shape of drugs, which exercise a special physiological influence on the system, or in whatever form, are useful only so far as they may excite, assist, or promote these natural curative processes.

4. That it should be the aim of the physician (after he has sedulously studied the clinical history of disease, and made him self master of its diagnosis), to inquire minutely into the intimate nature of these curative processes—their physiology, so to speak; to discover the best means of assisting them, to search for antidotes to morbid poisons, and to ascertain the best and most convenient methods of upholding vital power.

If one may venture a suggestion respecting the future of pathology, and of practice founded on it, it would be that a time is not far distant when all men who practice medicine in a scientific spirit, and divested of all the trammels of routine, will discard the distinction of acute inflammations and acute disease in general, into *asthenic* and *sthenic*—that all these maladies will be regarded as more or less asthenic, and as promoting more or less an undue waste of tissue, and that, in treatment, an object of primary importance will be the early adoption of means to uphold vital power, and the watchful and continued use of them throughout the duration of the case."

These propositions contain some truisms, some sophisms, and some glaring errors. For instance, the assertion that "disease is cured by natural processes," is a mere truism; for certainly it could not be cured by *unnatural* processes. Again, the assertion that remedies "are useful only so far as they excite, assist, or promote these natural curative processes" is purely sophistical; and when analized, amounts simply to the assertion that a remedy to act as such in the treatment of disease, must be capable of influencing in some way the natural curative processes capable of occurring in the animal economy. The second proposition quoted is entirely erroneous, as the author himself fully proves before he gets through his second lecture. Thus, on page 45, when speaking of the treatment of rheumatic fever, he says: "The seventh and last mode of treatment that I shall mention to you, is that which you have seen me adopt frequently at this Hospital, namely, *the treatment by elimination.* I give it this name in order that you may keep well in view its main object—to promote the *elimination* of morbid matter by the various emunctories, etc.". Again, on page 47, he says: "You perceive that all the means employed in this mode of treatment *tend to elimination*, and to the relief of pain; the opiate sudorific affecting the skin; the nitre and alkaline salts acting on the kidneys; the purgatives on the mucous membrane of the bowels; the wool and blisters on the joints." Now, inasmuch as this mode of treatment is the favorite one of the author, and he has reported numerous cases "cured" by it, without claiming that a single one of the remedies employed acted either as an "antidote," or as a "neutralizer of poison," we need go no further to prove the entire fallacy of his second proposition.

For what more "*direct influence*" can a remedy exert than that of a sudorific on the skin, a diuretic on the kidneys, or a cathartic on the bowels? Yet no one will pretend, that causing a direct *elimination* of morbid material, is either *antidoting* or *neutralizing* it. The first proposition we have quoted, declaring the notion that acute diseases are "cured by means which depress and reduce vital and nervous power," to be "altogether fallacious;" and the first clause of the third proposition, claiming that to promote the cure of disease by natural processes, "*vital power* must be upheld," affords a key to the whole book. Indeed they present the fundamental idea which the several lectures are simply designed to amplify and illustrate. In seeking for the exact meaning which the author intends to convey by *means* that depress *vital* power, we readily find that they are such as have long been included under the general term, *antiphlogistic* measures. And he expresses his decided belief, that the lectures contained in this volume, embody sufficient evidence to show that these means are "unnecessary for the cure of acute internal inflammations." On the other hand, the means for *upholding vital power*, are food, tonics, and so-called stimulants, of which alcohol is the chief. In conformity with these ideas, he plainly indicates his belief that all acute diseases are *asthenic* in their nature; and that "alcohol, in some form or other, is a remedy whose value can scarcely be over-estimated, and one upon which, when carefully administered, he relies with the utmost confidence in a great number of cases of disease which are at all amenable to treatment."

Such is a brief, but true statement of the doctrines of the learned and distinguished author of these lectures. Their importance, as indicating the present tendencies of a highly respectable portion of the professional mind, render them worthy of a careful examination. This we have neither space nor time to do at present. We will not leave the subject, however, without asking a few questions for the consideration of our readers.

1st. Are these doctrines true? Are the acute phlegmasia always *asthenic* in their nature? When we come to a patient with a florid flush in the face; a hot skin; hurried respiration; a full, strong, firm pulse; acute pain in the side, head, or joints; and acuteness of sensibility generally, are we to credit the evidence of our senses, and call it a case of active *sthenic* disease; or shall we ignore the evidence of our own senses, together with the clinical observations of the most extensive practitioners since the days of Hippocrates, and place such a case in the same catagory with one presenting a dingy flush on the face; a soft, compressible and frequent pulse; dull pain; and dulness of mental action? If the latter, then we might as well abandon all idea of making practical distinctions at the bed-side of the living patient. That most of the diseases seen by Dr. Todd during his long and honored service in King's College Hospital, have been *asthenic*, we have no doubt. But is he quite safe in considering the specific character of diseases in a London Hospital as fairly representing the character of disease everywhere? During the ten years that we have been connected with the Mercy Hospital of this city, we have not met with more than two or three of its inmates who presented such symptoms of an active sthenic condition, as to require the use of venesection or strong sedatives. But during the ten years that we practiced medicine in a rugged, hilly district in the interior of New York, we seldom met with a well marked case of pleurisy, pneumonia, arachnitis, or acute rheumatism, in the treatment of which, depletion and sedatives could be omitted without jeapording the life of the patient. Have our senses and the results of our practice alike deceived us all through life? Or can we rely on it as a settled fact in pathology, that the specific or particular character of diseases vary with the variations of locality, climate, season, temperament, and other circumstances attendant on their development?

The reader of the volume before us would be more likely to adopt the theories of its author, if the treatment of disease recommended in it was more consistent with such theories. For while the author intimates that all acute diseases are asthenic, and that the idea of curing them by antiphlogistic means is entirely fallacious, he treats the very first disease chosen to illustrate his pathological views, with anodyne sudorifics, diuretics, and purgatives ; remedies which have certainly been ranked as antiphlogistic since the days of Hippocrates. And if sweating, diuresis and purging are not positively depletive, it is quite time that we altered the whole phraseology of our Materia Medica.

2. What does our author mean by " *Vital Power* " ? Is it the *vis anima* or *vis vitæ* of the older writers ? Is it the *vis medicatrix naturæ* of the modern ? Or is it an undefined shadowy something that rules like a presiding genius over the various organic movements and functions of the human system ? We fully agree with Dr. Todd, that the prevalent doctrines of pathology and therapeutics, should be carefully reviewed : and we think one of the first and most important objects of such review should be, either to expunge from our literature, or definitely define all such words and phrases as *vital power*, vital force, nervous force, vis medicatrix, and others used to conceal ignorance, or to express ideas too vague to be understood. If the pathologist tells us that the blood is capable of being altered in the relative proportion of its constituents, or may have new matters introduced into it, thereby causing diseases which are to be cured by remedies that either increase the deficient constituents, or neutralize or expel the foreign matters, we can understand fully his meaning. If he tells us that all living organized structures necessarily possess a *property* by which the primary cells, or particles of which they are composed, are made to assume a certain definite relation to each other, and which property he calls *vital affinity*, we can understand readily his meaning. If he tells us that all living organized matter possesses another property, by which it is rendered capable of being acted upon by exterior agents, and which he calls *susceptibility*, we can attach a well defined idea to his language. If he goes further, and informs us that every structure has its elementary function, such as secretion for the secreting cells; contraction for the muscular structure; sensibility and transmissability for the nervous, etc., he can be understood. If he tells us that any one of these properties or elementary functions, separately, or all of them unitedly, may be increased, diminished, or perverted, constituting elementary forms of morbid action, out of which the complex phenomena of disease are developed; and that curative measures must be sought in such agents as will depress these properties and functions when excited; increase them when depressed; or correct them when perverted, we can comprehend the meaning of his language, and grasp clearly the ideas he wishes to convey. But when he talks of sustaining or depressing *vital power*, or nervous power, or the *vis medicatrix naturæ*, we will not say he talks nonsense; but he certainly talks of something very imperfectly defined.

2. What are the *natural curative processes?*

Within a few years past, much has been written by Drs. Forbes, Bigelow, and their disciples, about the "natural curative processes," "nature in the cure of disease," etc. We are gravely assured that it is the office of the physician *not to cure* disease, but only to *aid nature* in affecting a cure.

It is very easy to sit down at ones desk and magnify the sanitary powers of dame nature; and write many pretty things about her wonderful accomplishments. But after all, we are very much inclined to think that it would be far more satisfactory to the anxious, toiling practitioner, at the bed-side of his patient, to be informed definitely what *nature* is? What the *natural curative processes?* And by what means they can be most readily influenced in any given direction.

We seldom read anything on this subject without thinking of a discussion in a Medical Society, to which we had the pleasure of listening. The subject under consideration was the treatment of an acute and serious disease. One member rose and said that he considered the disease as specific in its nature, destined to run a given course, and seldom or never needed any treatment at all; and expressed his very decided conviction that if every case was left entirely to good nursing, the ratio of mortality from the disease would be very much less. He likened the disease to an engine running on a Rail Road track, and the business of the physician was simply to keep the *track clear.* When questioned, however, in relation to the liability of having obstructions on the track, and the best means for their removal, he enumerated under the first head almost all the consequences or important morbid developments of the disease, and under the latter an array of medicinal agents more numerous and active than we had even thought of using in the same form of disease. Would not the comparison have been better if the Doctor had made the engine represent *nature*, or more properly, the living, healthy human system, and the obstruction on the track, the disease? For what is disease but a deviation from health, an obstruction to the healthy performance of one or more of the natural processes and functions of the system? And in what does the cure of disease consist, except in the removal of such obstructions or deviations from healthy action?

We had intended to notice the last lecture in this volume, which discusses the therapeutic relations of alcohol; and which we think presents many important errors. But we have occupied very much more space than usual already, and consequently will not pursue the subject further at present.

All the above-named works were received from the publishers, through the extensive Book Establishment of S. C. Griggs & Co., of this city.

We have also received from the Author, a volume entitled "Mal-practice and Medical Evidence," which will be noticed in our next issue.

